# Phase I study of napabucasin in combination with FOLFIRI + bevacizumab in Japanese patients with metastatic colorectal cancer

**DOI:** 10.1007/s10147-021-01987-9

**Published:** 2021-07-21

**Authors:** Hiroya Taniguchi, Toshiki Masuishi, Akihito Kawazoe, Kei Muro, Shigenori Kadowaki, Hideaki Bando, Shuichi Iino, Rie Kageyama, Takayuki Yoshino

**Affiliations:** 1 Department of Gastrointestinal Oncology, National Cancer Center Hospital East, Chiba, Japan; 2 Department of Clinical Oncology, Aichi Cancer Center Hospital, 1-1, Kanokoden, Chikusaku, Nagoya, 464-8681 Japan; 3 Sumitomo Dainippon Pharma Co., Ltd, Osaka, Japan

**Keywords:** Colorectal cancer, Napabucasin, FOLFIRI, Phase I

## Abstract

**Background:**

Napabucasin is an oral NAD(P)H:quinone oxidoreductase 1 bioactivatable agent that generates reactive oxygen species, is hypothesised to affect multiple oncogenic cellular pathways, including STAT-3, and is expected to result in cancer cell death. This phase I study investigated the safety, tolerability, and pharmacokinetics of napabucasin co-administered with fluorouracil, *l*-leucovorin, and irinotecan (FOLFIRI) chemotherapy plus bevacizumab in Japanese patients with metastatic colorectal cancer (CRC).

**Methods:**

Patients with histologically confirmed unresectable stage IV CRC received oral napabucasin 240 mg twice daily (BID). Intravenous FOLFIRI and bevacizumab therapy was initiated on day 3 at approved doses. Unacceptable toxicity was evaluated over the first 30 days of treatment, after which treatment continued in 14-day cycles until toxicity or disease progression. Endpoints included safety, pharmacokinetics, and tumour response based on RECIST v1.1.

**Results:**

Four patients received treatment; three were evaluable during the unacceptable toxicity period. All four patients experienced diarrhoea and decreased appetite (considered napabucasin-related in four and two patients, respectively), and three patients experienced neutrophil count decreased. No unacceptable toxicity was reported during the 30-day evaluation period. No grade 4 events, deaths, or serious adverse events were reported. The addition of FOLFIRI and bevacizumab to napabucasin did not significantly change the pharmacokinetic profile of napabucasin; however, results were variable among patients. The best overall response was stable disease in two patients (50.0%).

**Conclusions:**

Napabucasin 240 mg BID in combination with FOLFIRI and bevacizumab was tolerated, with a manageable safety profile in Japanese patients with metastatic CRC.

**Supplementary Information:**

The online version contains supplementary material available at 10.1007/s10147-021-01987-9.

## Introduction

Colorectal cancer (CRC) mortality continues to increase in Japan, with more than 50,000 deaths occurring in 2016 [1]. Primary treatment of CRC consists of either surgical resection of tumours or systemic chemotherapy for unresectable CRC [2]. Commonly used chemotherapy regimens include fluorouracil, *l*-leucovorin, and oxaliplatin (FOLFOX) or irinotecan (FOLFIRI) with or without molecular-targeted agents [1]. However, therapeutic success with these options remains limited [1].

Accumulating evidence indicates that cancer stem cells (CSCs) are a highly malignant sub-population of cancer cells with self-renewal capability that play a role in the pathogenesis of CRC, including malignant growth, recurrence, drug resistance, and metastasis [3]. CSCs isolated from patients with CRC display tumour-initiating properties as well as resistance to chemotherapies [4, 5]. As such, CSC inhibitors are considered to be a novel strategy for the treatment of CRC. It could be that an approach that combines CSC inhibition with standard treatments may offer improved effectiveness and outcomes in patients with CRC.

Napabucasin is an oral NAD(P)H:quinone oxidoreductase 1 (NQO1) bioactivatable agent that generates reactive oxygen species; is hypothesized to affect multiple oncogenic cellular pathways, including signal transducer and activator of transcription 3 (STAT-3); and is expected to result in cancer cell death [6]. In preclinical studies, napabucasin inhibited self‐renewal and downregulated markers of stemness, leading to apoptosis of CSC [7, 8]. Encouraging signs of anti-cancer activity were observed in pretreated patients with advanced CRC in North America, including in patients previously treated with FOLFIRI with or without bevacizumab [9]. This phase I study investigated the safety, tolerability, and pharmacokinetics of napabucasin when co-administered with FOLFIRI plus bevacizumab in Japanese patients with metastatic CRC.

## Patients and methods

### Patients and study design

This multi-centre, open-label, phase I study in patients with metastatic CRC evaluated the safety and tolerability of napabucasin with FOLFIRI and bevacizumab and examined the pharmacokinetic profile and exploratory anti-tumour activity of this combination (NCT02641873). Eligible patients included those: with histologically confirmed unresectable stage IV CRC, irrespective of previous chemotherapy and *KRAS* mutation status; aged at least 20 years, with a life expectancy of at least 3 months; with an Eastern Cooperative Oncology Group performance status of 0 or 1; and who met predetermined criteria for main organ function. Key exclusion criteria included patients who: had received chemotherapy, radiotherapy, hormonal therapy, immunotherapy, or thermotherapy for the primary disease within 21 days of enrolment; had undergone major surgery within 28 days of enrolment; had brain metastasis that required treatment or was symptomatic; had multiple primary cancers at the time of enrolment; had carcinomatous pleural effusion, ascites, or pericardial effusion requiring invasive treatment; had Crohn’s disease or ulcerative colitis, a history of extensive resection of the small intestine, diarrhoea, or intestinal paralysis or intestinal obstruction; had concurrent gastrointestinal perforation, tracheoesophageal fistula, or severe fistula; or had uncontrollable concurrent diseases.

**Fig. 1:**
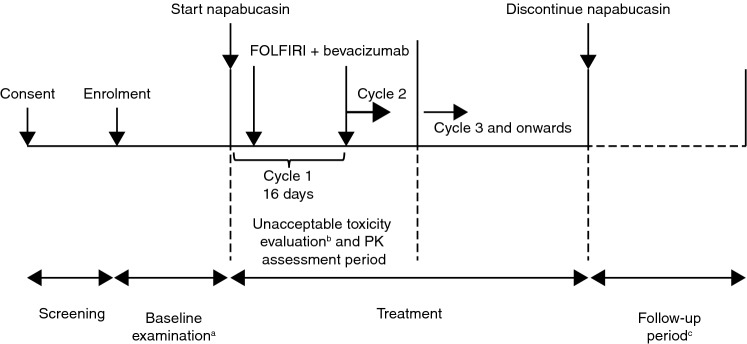
Study flowchart. *PK* pharmacokinetic. ^a^Examination ≤ 7 days before the start of napabucasin. ^b^30-day assessment. ^c^28-day follow-up from the last dose of napabucasin

On day 1, patients in a fasted state received only one dose of napabucasin; thereafter, napabucasin was administered orally at a dose of 240 mg twice daily. FOLFIRI was administered as *l*-leucovorin 200 mg/m^2^ intravenous infusion over at least 2 h started concurrently with irinotecan 180 mg/m^2^ intravenous infusion over at least 1.5 h; once *l*-leucovorin administration was completed, fluorouracil 400 mg/m^2^ was administered by bolus injection followed by continuous infusion at a dose of 2400 mg/m^2^ over 46–48 h. Bevacizumab 5 mg/kg was administered as a 90-min intravenous infusion. FOLFIRI and bevacizumab therapy was started on day 3. From cycle 2 onward, each cycle of FOLFIRI and bevacizumab therapy consisted of 14 days, with administration of FOLFIRI and bevacizumab initiated on day 1 (Fig. 1).

Unacceptable toxicity was evaluated during the ‘unacceptable toxicity evaluation period’, which was defined in the protocol as the first 30 days of treatment (Fig. 1). After the unacceptable toxicity evaluation period, napabucasin in combination with FOLFIRI and bevacizumab was to be continued until intolerable adverse events (AEs) or progression of the primary disease and AEs were evaluated during the entire study period.

Three patients were to be initially enrolled in the study (Online Resource 1). If none of the patients experienced unacceptable toxicity during the unacceptable evaluation period, enrolment was to be terminated and napabucasin in combination with FOLFIRI and bevacizumab would be deemed well tolerated in Japanese patients. If one patient experienced unacceptable toxicity, an additional three patients would be enrolled, for a total of six patients. If two of three patients experienced unacceptable toxicity, the study sponsor would decide whether to add three more patients based on discussion with the coordinating investigator and the Independent Data Monitoring Committee, if needed. If one of six enrolled patients experienced unacceptable toxicity, enrolment was to be terminated and napabucasin in combination with FOLFIRI and bevacizumab would be deemed well tolerated in Japanese patients. If two of six patients experienced unacceptable toxicity, the sponsor would discuss with the coordinating investigator, with input from the Independent Data Monitoring Committee, to determine whether napabucasin in combination with FOLFIRI and bevacizumab was considered tolerated. Unacceptable toxicity in all three initially enrolled patients or in three or more of six enrolled patients would indicate that the combination was not tolerated, and the study would be terminated.

Napabucasin in combination with FOLFIRI and bevacizumab was continued until intolerable AEs or progression of the primary disease. If neither fluorouracil nor irinotecan was able to be administered at any point, napabucasin therapy was discontinued. A 28-day follow-up period commenced after the last dose of napabucasin.

All patients provided written informed consent before enrolment and study methodologies conformed to the standards set by the Declaration of Helsinki and were approved by the appropriate ethics committee at each study site. All procedures were conducted in accordance with the ethical standards of the institutional and/or national research committee at each study site and with the 1964 Helsinki declaration and its later amendments or comparable ethical standards.

### Study endpoints and assessments

Safety endpoints included assessment of AEs and unacceptable toxicity, vital signs, body weight, laboratory values, and 12-lead electrocardiogram. AEs were coded using the Medical Dictionary for Regulatory Activities version 18.0 and graded according to the Common Terminology Criteria for Adverse Events v4.03. Unacceptable toxicity was assessed as any of the following events occurring in the first 30 days of treatment and considered to be related to napabucasin: grade 4 neutropenia persisting for ≥ 8 days despite appropriate treatment; febrile neutropenia persisting for ≥ 5 days despite appropriate treatment; grade 3 thrombocytopenia requiring platelet transfusion and grade 4 thrombocytopenia; anorexia, nausea, and vomiting for ≥ 4 days after the onset of grade 3 toxicity that does not improve to grade ≤ 2 or resolve after appropriate treatment; diarrhoea for ≥ 6 days after the onset of grade 3 toxicity that does not improve to grade ≤ 2 or resolve after appropriate treatment; other grade ≥ 3 non-haematological toxicities, excluding grade 3 or 4 electrolyte abnormality determined to be less clinically significant and grade 3 hypertension controllable with appropriate treatment; or other events considered to be clinically important.

Pharmacokinetic analysis of plasma napabucasin occurred pre-dose and 2, 4, 6, 8, 10, 12, and 24 h post-dose on days 1 and 30, pre-dose, and 12 and 24 h post-dose on day 3, and pre-dose on days 17 or 24 following initiation of napabucasin treatment. Plasma irinotecan was measured on day 3 immediately before and after irinotecan dosing and 12 h after napabucasin dosing, and plasma fluorouracil concentrations were measured at one point immediately before or after irinotecan dosing or immediately before fluorouracil dosing on day 3, and immediately after and 1 h after fluorouracil dosing on day 5. Plasma concentrations of unchanged napabucasin, fluorouracil, irinotecan, and SN-38 (an active metabolite of irinotecan) were calculated at each respective evaluation time point. In addition, the pharmacokinetic parameters of napabucasin were calculated using the plasma concentration of unchanged napabucasin.

Response was evaluated using computed tomography or magnetic resonance imaging at baseline and every 8 weeks after initiation of napabucasin using Response Evaluation Criteria in Solid Tumors (RECIST) version 1.1 [10]. Progression-free survival was defined as the time from the start of treatment to documented progressive disease as assessed by RECIST version 1.1 or death, whichever came earlier. Progression-free survival was evaluated for 12 months after initiation of napabucasin in the last patient enrolled.

### Statistical analysis

The safety analysis population included all patients who received any dose of napabucasin. Unacceptable toxicity was evaluated in those patients who received at least 70% of the planned dose of napabucasin during the first 30 days of treatment in addition to the planned doses of FOLFIRI and bevacizumab, or patients who developed unacceptable toxicity regardless of napabucasin compliance rate or FOLFIRI and bevacizumab therapy status. Pharmacokinetics were evaluated in patients who received napabucasin and had post-dose plasma napabucasin concentration data for at least one time point. Summary statistics were calculated where appropriate. Progression-free survival was calculated by the Kaplan–Meier method.

## Results

### Patients

Overall, four patients were enrolled in the study. During the unacceptable toxicity evaluation period, one patient withdrew their consent to participate in the study, prompting the enrolment of one additional patient as per protocol. Three patients were female (75.0%), with a median age of 54.0 years (range 46–65); Eastern Cooperative Oncology Group performance status was 0 in all patients (Table 1). All four patients had received prior chemotherapy (three [75.0%] had received FOLFIRI plus bevacizumab and two [50.0%] had received leucovorin, fluorouracil, oxaliplatin, and irinotecan plus bevacizumab) and three patients (75.0%) had received prior surgery. The patient who withdrew from the study during the unacceptable toxicity evaluation period received treatment with napabucasin in combination with FOLFIRI and bevacizumab for 14 days (less than one cycle), while the three patients who completed the unacceptable toxicity evaluation period received treatment for 51–338 days (3–20 cycles). Study discontinuation in the three patients who completed the unacceptable toxicity evaluation period was due to progressive disease. Overall, treatment compliance for napabucasin throughout the study period was 90–100%.

**Table 1:** Patient demographics and baseline characteristics

	Total (*n* = 4)
Female, *n* (%)	3 (75.0)
Median age, years (range)	54.0 (46–65)
Eastern Cooperative Oncology Group performance status, *n* (%)	
0	4 (100)
1	0
Primary cancer, *n* (%)	
Colon	4 (100)
Rectum	0
Median duration of disease, years (range)	1.4 (1–3)
Concurrent diseases and symptoms associated with the primary disease, *n* (%)	
Yes	4 (100)
No	0
Prior anti-cancer therapies, *n* (%)	
Drug therapy	4 (100)
Surgery	3 (75.0)
Radiotherapy	0
Number of regimens of prior drug therapy, *n* (%)	
1	1 (25.0)
2	2 (50.0)
≥ 3	1 (25.0)
Prior treatment with FOLFIRI plus bevacizumab	3 (75.0)
Prior treatment with FOLFOXIRI plus bevacizumab	2 (50.0)

*FOLFIRI* fluorouracil, *l*-leucovorin, and irinotecan, *FOLFOXIRI* fluorouracil, *l*-leucovorin, oxaliplatin, and irinotecan

### Safety

Treatment-emergent AEs were reported in all four patients in the full safety analysis population. All four patients experienced diarrhoea and decreased appetite, and three patients experienced neutrophil count decreased (Table 2, Online Resource 2). In three patients who were evaluable for the unacceptable toxicity population, no events classified as unacceptable toxicity were reported during the evaluation period (Online Resource 3). All four cases of diarrhoea and two cases of decreased appetite were considered related to napabucasin. Loperamide was used in all patients who had diarrhoea. Most AEs, including gastrointestinal disorders, were of grade 1 or 2, with most events occurring during the first 9 days of treatment. Overall, there were two cases of grade 3 neutrophil count decreased, not considered related to napabucasin. No grade 4 events, deaths, or serious AEs were reported.

**Table 2:** Total number of adverse events of any grade occurring in the safety analysis population

*n* (%)	Total (*n* = 4)
Any adverse event^a^	4 (100)
Gastrointestinal disorders	4 (100)
Diarrhoea	4 (100)
Nausea	2 (50.0)
Stomatitis	2 (50.0)
Abdominal pain	1 (25.0)
Periodontal disease	1 (25.0)
Vomiting	1 (25.0)
Anal inflammation	1 (25.0)
Metabolism and nutrition disorders	4 (100)
Decreased appetite	4 (100)
Investigations	3 (75.0)
Neutrophil count decreased	3 (75.0)
Blood bilirubin increased	1 (25.0)
Platelet count decreased	1 (25.0)
General disorders and administration site conditions	2 (50.0)
Malaise	2 (50.0)
Peripheral oedema	1 (25.0)
Infections and infestations	1 (25.0)
Nasopharyngitis	1 (25.0)
Musculoskeletal and connective tissue disorders	1 (25.0)
Muscle spasms	1 (25.0)
Nervous system disorders	1 (25.0)
Cholinergic syndrome	1 (25.0)
Psychiatric disorders	1 (25.0)
Insomnia	1 (25.0)
Renal and urinary disorders	1 (25.0)
Chromaturia	1 (25.0)
Proteinuria	1 (25.0)
Respiratory, thoracic, and mediastinal disorders	1 (25.0)
Epistaxis	1 (25.0)
Hiccups	1 (25.0)
Skin and subcutaneous tissue disorders	1 (25.0)
Dry skin	1 (25.0)
Skin hyperpigmentation	1 (25.0)
Vascular disorders	1 (25.0)
Hypertension	1 (25.0)

^a^Adverse events were coded using the medical dictionary for regulatory activities version 18.0

Three patients (75.0%) experienced a total of seven AEs leading to interruption of napabucasin; diarrhoea in three patients and abdominal pain and malaise in one patient each. Periods of dose interruption ranged from 0.5 to 8.5 days, although it is difficult to associate dose-interruption duration with AE occurrence. One of the patients with diarrhoea also had a dose reduction of napabucasin. These events were all considered related to napabucasin, and all cases of diarrhoea and abdominal pain resolved. No patients discontinued napabucasin due to AEs. Two patients (50.0%) experienced AEs which led to interruption of FOLFIRI or bevacizumab; decreased neutrophil count in one patient, and decreased appetite, malaise, and stomatitis in one patient. One patient (25.0%) experienced AEs which led to dose reduction of FOLFIRI or bevacizumab therapy (nausea and decreased appetite).

### Pharmacokinetics

**Fig. 2:**
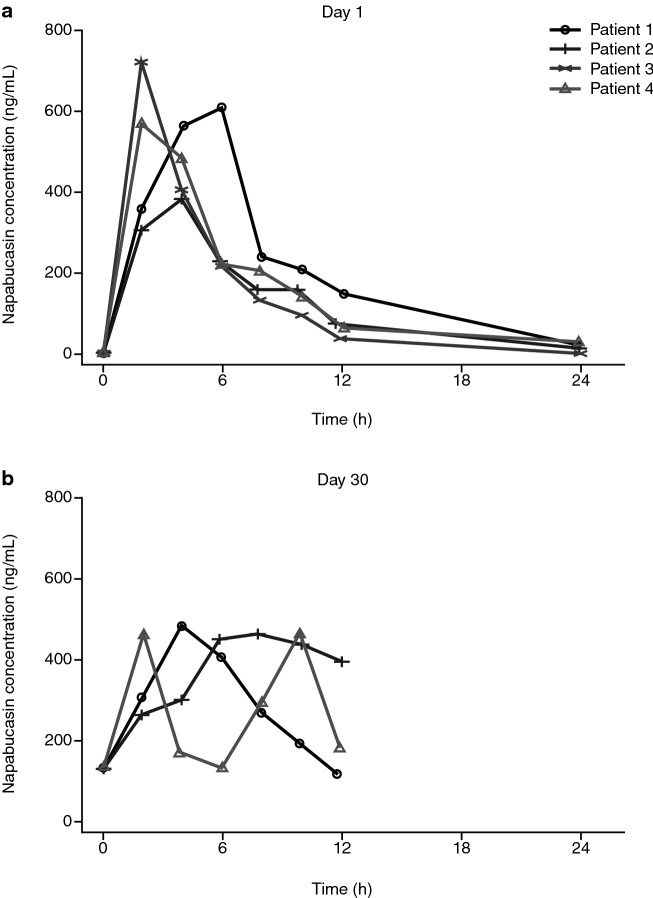
Plasma concentration of napabucasin on **a** day 1 and **b** day 30 for each patient (pharmacokinetic analysis population)

The time to maximum plasma concentration of napabucasin on day 1 was 2–6 h; however, profiles varied between patients (Fig. 2). Following twice-daily administration of napabucasin, the plasma concentration on day 30 was comparable with or slightly lower than the plasma concentration after single administration, with a bimodal profile observed in one patient (Fig. 2). On day 1, area under the curve from 0 to 12 h (AUC_0–12_) ranged from 2500 to 4118 ng·h/mL and the maximum concentration ranged from 381 to 720 ng/mL; on day 30, AUC_0–12_ ranged from 3542 to 4385 ng·h/mL and the maximum concentration ranged from 464 to 483 ng/mL (Online Resource 4).

The effects on the pharmacokinetics of napabucasin in combination with FOLFIRI and bevacizumab therapy were not distinct because of variation among patients (Fig. 2), suggesting no significant change. In addition, the trough value on day 3 (the first day of administration of napabucasin in combination with FOLFIRI and bevacizumab therapy) showed no substantial difference from that on day 30 for two of the three patients (data not shown).

The median plasma concentration of fluorouracil on day 5 was 1475.5 ng/mL for continuous intravenous infusion and 109.0 ng/mL 1 h after completion of infusion. The median plasma concentrations of irinotecan and SN-38 (an active metabolite of irinotecan) on day 3 were 1885.0 ng/mL and 21.2 ng/mL immediately after completion of the infusion, and 317.5 ng/mL and 0 ng/mL 12 h after napabucasin administration, respectively. Napabucasin exposure and adverse events for each patient are described in Table 3.

**Table 3:** Adverse events (AEs) leading to treatment interruption, maximum concentration (*C*_max_) and area under the curve from 0 to infinity (AUC_0–inf_) of napabucasin on days 1 and 30 for each patient

Patient identifier for the study	Day 1 *C*_max_ (ng/mL)	Day 30 *C*_max_ (ng/mL)	Day 1 AUC_0-inf_ (h ng/mL)	Day 30 AUC_0-inf_ (h ng/mL)	Duration of treatment (days)	List of AEs related to treatment	AEs leading to treatment interruption
30,001	609	483	5249	4064	51	Anorexia Malaise Diarrhoea	N/A
30,002	381	464	3082	N/A	220	Diarrhoea Urine discolouration Diarrhoea Diarrhoea Diarrhoea Diarrhoea Diarrhoea Diarrhoea Diarrhoea Diarrhoea	Diarrhoea
30,003	720	N/A	3275	N/A	5	Diarrhoea Abdominal pain	Diarrhoea Abdominal pain
30,004	569	467	4073	N/A	338	Anorexia Diarrhoea Diarrhoea Malaise Diarrhoea Diarrhoea	Diarrhoea Malaise

*N/A* not applicable

### Anti-tumour activity

The best overall response was stable disease in two patients (50.0%) and progressive disease in two patients (50.0%); the disease control rate was 50.0% (two of four patients) (Online Resource 5). Median progression-free survival was 4.4 months (range 0.79–11.1).

## Discussion

This phase I study evaluated the safety, tolerability, and exploratory anti-tumour activity of napabucasin in combination with FOLFIRI and bevacizumab in Japanese patients with metastatic CRC. Overall, napabucasin 240 mg twice daily in combination with FOLFIRI and bevacizumab was tolerable in this small population, with a manageable safety profile. This was evidenced by an excellent compliance rate (90–100%) for napabucasin treatment throughout the study period, with no patients discontinuing treatment due to AEs; 3 patients discontinued due to progressive disease and another patient discontinued due to withdrawal by the patient.

The most common AEs were diarrhoea, decreased appetite, and decreased neutrophil count. AEs were largely managed or reversed through dose interruption, dose reduction in one patient, or concomitant administration of loperamide. AEs detected in this study were similar to those observed in a phase III trial of napabucasin monotherapy in patients with refractory advanced CRC [11], as well as in studies of napabucasin in combination with paclitaxel in Japanese patients with advanced or recurrent gastric cancer [12] and in combination with FOLFIRI with or without bevacizumab in patients with metastatic CRC [9]. No substantial differences in the rate of onset or severity were identified compared with napabucasin monotherapy in Japanese patients [13], and no new clinically important AEs were observed.

Plasma concentration of napabucasin at day 1 peaked about 2–6 h after administration and was quickly eliminated; however, results were highly variable between patients. The high inter-patient variability observed here is similar to that reported in other studies of napabucasin [12, 13]*.* Plasma concentration with twice-daily dosing remained similar to that of the single dose administered on day 1. The effect of concomitant FOLFIRI and bevacizumab on the pharmacokinetics of napabucasin appeared to show no apparent pharmacokinetic interactions. While there were some adverse events considered related to napabucasin, there was no clear correlation between napabucasin exposure and rate of adverse events.

Overall, this study showed that napabucasin 240 mg twice daily in combination with FOLFIRI and bevacizumab therapy was tolerable in Japanese patients with similar pharmacokinetic parameters. However, the small sample size of this study makes it difficult to draw meaningful conclusions, and additional validation of these results is needed. A phase III trial is currently evaluating napabucasin in combination with FOLFIRI in patients with previously treated metastatic CRC (NCT02753127), which will include the enrolment of Japanese patients.

## Supplementary Information

Below is the link to the electronic supplementary material.

Supplementary file1 (DOCX 19 KB)

Supplementary file2 (EPS 2033 KB)

Supplementary file3 (EPS 1967 KB)

## Data Availability

The datasets generated during and/or analysed during the current study are from a limited population (*n* = 4); therefore, data cannot be shared to ensure appropriate anonymity of patients.
