# Supplementary material for: Phase I study of napabucasin in combination with FOLFIRI + bevacizumab in Japanese patients with metastatic colorectal cancer
Source: Int J Clin Oncol. 2021 Jul 21;26(11):2017–24. doi: 10.1007/s10147-021-01987-9 (PMC8520863; doi:10.1007/s10147-021-01987-9)
Supplement: Supplementary file 1 — Supplementary file1 (DOCX 19 KB) [file 10147_2021_1987_MOESM1_ESM.docx]

**Online resources**

**Phase I study of napabucasin in combination with FOLFIRI + bevacizumab in Japanese patients with metastatic colorectal cancer**

Hiroya Taniguchi^1,2^ **·** Toshiki Masuishi^2^ **·** Akihito Kawazoe^1^  **·** Kei Muro^2^ **·** Shigenori Kadowaki^2^ **·** Hideaki Bando^2^ **·** Shuichi Iino^3^ **·** Rie Kageyama^3^ **·** Takayuki Yoshino^1^

^1^Department of Gastrointestinal Oncology, National Cancer Center Hospital East, Chiba, Japan

^2^Department of Clinical Oncology, Aichi Cancer Center Hospital, Nagoya, Japan

^3^Sumitomo Dainippon Pharma Co., Ltd, Osaka, Japan

**Online Resource 1** Flowchart of additional patients to assess tolerability. ^a^If unacceptable toxicity occurred in 2 of 3 patients, the sponsor and coordinating investigator discussed and decided whether to add 3 additional patients or assess tolerability without adding 3 additional patients. The sponsor sought opinions from the Independent Data Monitoring Committee

**Online Resource 2** Adverse events by grade occurring in the safety analysis population

| ***n* (%)** | **Grade 1** | **Grade 2** | **Grade 3** | **Grade 4** | **Grade 5** | **Total (*n* = 4)** |
| --- | --- | --- | --- | --- | --- | --- |
| Any adverse event^a^ | 0 | 2 (50.0) | 2 (50.0) | 0 | 0 | 4 (100) |
| Decreased appetite | 3 (75.0) | 1 (25.0) | 0 | 0 | 0 | 4 (100) |
| Diarrhoea | 1 (25.0) | 3 (75.0) | 0 | 0 | 0 | 4 (100) |
| Neutrophil count decreased | 0 | 1 (25.0) | 2 (50.0) | 0 | 0 | 3 (75.0) |
| Malaise | 1 (25.0) | 1 (25.0) | 0 | 0 | 0 | 2 (50.0) |
| Nausea | 1 (25.0) | 1 (25.0) | 0 | 0 | 0 | 2 (50.0) |
| Stomatitis | 0 | 2 (50.0) | 0 | 0 | 0 | 2 (50.0) |
| Abdominal pain | 1 (25.0) | 0 | 0 | 0 | 0 | 1 (25.0) |
| Anal inflammation | 1 (25.0) | 0 | 0 | 0 | 0 | 1 (25.0) |
| Blood bilirubin increased | 1 (25.0) | 0 | 0 | 0 | 0 | 1 (25.0) |
| Cholinergic syndrome | 1 (25.0) | 0 | 0 | 0 | 0 | 1 (25.0) |
| Chromaturia | 1 (25.0) | 0 | 0 | 0 | 0 | 1 (25.0) |
| Dry skin | 1 (25.0) | 0 | 0 | 0 | 0 | 1 (25.0) |
| Epistaxis | 1 (25.0) | 0 | 0 | 0 | 0 | 1 (25.0) |
| Hiccups | 1 (25.0) | 0 | 0 | 0 | 0 | 1 (25.0) |
| Hypertension | 0 | 1 (25.0) | 0 | 0 | 0 | 1 (25.0) |
| Insomnia | 1 (25.0) | 0 | 0 | 0 | 0 | 1 (25.0) |
| Muscle spasms | 1 (25.0) | 0 | 0 | 0 | 0 | 1 (25.0) |
| Nasopharyngitis | 1 (25.0) | 0 | 0 | 0 | 0 | 1 (25.0) |
| Periodontal disease | 0 | 1 (25.0) | 0 | 0 | 0 | 1 (25.0) |
| Peripheral oedema | 1 (25.0) | 0 | 0 | 0 | 0 | 1 (25.0) |
| Platelet count decreased | 1 (25.0) | 0 | 0 | 0 | 0 | 1 (25.0) |
| Proteinuria | 1 (25.0) | 0 | 0 | 0 | 0 | 1 (25.0) |
| Skin hyperpigmentation | 0 | 1 (25.0) | 0 | 0 | 0 | 1 (25.0) |
| Vomiting | 1 (25.0) | 0 | 0 | 0 | 0 | 1 (25.0) |

^a^Adverse events were coded using the Medical Dictionary for Regulatory Activities version 18.0

**Online Resource 3** Adverse events by grade during the unacceptable toxicity evaluation period (unacceptable toxicity population)

| ***n* (%)** | **Grade 1** | **Grade 2** | **Grade 3** | **Grade 4** | **Grade 5** | **Total  (*n* = 3)** |
| --- | --- | --- | --- | --- | --- | --- |
| Any adverse event^a^ | 0 | 2 (66.7) | 1 (33.3) | 0 | 0 | 3 (100) |
| Decreased appetite | 2 (66.7) | 1 (33.3) | 0 | 0 | 0 | 3 (100) |
| Diarrhoea | 2 (66.7) | 1 (33.3) | 0 | 0 | 0 | 3 (100) |
| Decreased neutrophil count | 0 | 1 (33.3) | 1 (33.3) | 0 | 0 | 2 (66.7) |
| Stomatitis | 1 (33.3) | 1 (33.3) | 0 | 0 | 0 | 2 (66.7) |
| Anal inflammation | 1 (33.3) | 0 | 0 | 0 | 0 | 1 (33.3) |
| Chromaturia | 1 (33.3) | 0 | 0 | 0 | 0 | 1 (33.3) |
| Hiccups | 1 (33.3) | 0 | 0 | 0 | 0 | 1 (33.3) |
| Hypertension | 0 | 1 (33.3) | 0 | 0 | 0 | 1 (33.3) |
| Insomnia | 1 (33.3) | 0 | 0 | 0 | 0 | 1 (33.3) |
| Malaise | 1 (33.3) | 0 | 0 | 0 | 0 | 1 (33.3) |
| Nausea | 0 | 1 (33.3) | 0 | 0 | 0 | 1 (33.3) |
| Vomiting | 1 (33.3) | 0 | 0 | 0 | 0 | 1 (33.3) |

^a^Adverse events were coded using the Medical Dictionary for Regulatory Activities version 18.0

**Online Resource 4** (**a**) Maximum concentration (*C*_max_) and (**b**) area under the curve from 0 to 12 hours (AUC_0–12_) of napabucasin on days 1 and 30 for each patient (pharmacokinetic analysis population)

**Online Resource 5**  Best overall response

| ***n* (%)** | **Total (*n* = 4)** |
| --- | --- |
| Best overall response |  |
| Complete response | 0 |
| Partial response | 0 |
| Stable disease | 2 (50.0) |
| Progressive disease | 2 (50.0) |
| Objective response rate | 0 |
| Disease control rate | 2 (50.0) |
